# Quality Indicators for Colonoscopy Procedures: A Prospective Multicentre Method for Endoscopy Units

**DOI:** 10.1371/journal.pone.0033957

**Published:** 2012-04-11

**Authors:** Romain Coriat, Augustin Lecler, Dominique Lamarque, Jacques Deyra, Hervé Roche, Catherine Nizou, Olivier Berretta, Bruno Mesnard, Martin Bouygues, Alain Soupison, Jean-Luc Monnin, Philippe Podevin, Carole Cassaz, Denis Sautereau, Frédéric Prat, Stanislas Chaussade

**Affiliations:** 1 Service de Gastroentérologie, Hôpital Cochin, GHU Ouest, Paris, France; 2 Université Paris Descartes, Paris, France; 3 Service de Gastroentérologie, Hôpital Hotel-Dieu, GHU Ouest, Paris, France; 4 Unité de recherche clinique, URC Cochin, Paris France; 5 Hôpital privé de Versailles, Site Château de la Maye, Versailles, France; 6 Service de Gastroentérologie, Hôpital d'instruction des armées Bégin, Saint Mandé, France; 7 Clinique chirurgicales de Chelles, Chelles, France; 8 Service de Gastroentérologie, Centre hospitalier de Tourcoing, Tourcoing, France; 9 Clinique du Pré, Le mans, France; 10 Service de Gastroentérologie, Centre Hospitalier William Morey, Chalon-sur-Saône, France; 11 Clinique Medico Chirurgicale, St Jean-de-Vedas, France; 12 Service de Gastroentérologie, Centre hospitalier de Provins, Provins, France; 13 Service de Gastroentérologie, Hôpital universitaire, Limoges, France; Technische Universität München, Germany

## Abstract

**Background and Aims:**

Healthcare professionals are required to conduct quality control of endoscopy procedures, and yet there is no standardised method for assessing quality. The topic of the present study was to validate the applicability of the procedure in daily practice, giving physicians the ability to define areas for continuous quality improvement.

**Methods:**

In ten endoscopy units in France, 200 patients per centre undergoing colonoscopy were enrolled in the study. An evaluation was carried out based on a prospectively developed checklist of 10 quality-control indicators including five dependent upon and five independent of the colonoscopy procedure.

**Results:**

Of the 2000 procedures, 30% were done at general hospitals, 20% at university hospitals, and 50% in private practices. The colonoscopies were carried out for a valid indication for 95.9% (range 92.5–100). Colon preparation was insufficient in 3.7% (range 1–10.5). Colonoscopies were successful in 95.3% (range 81–99). Adenoma detection rate was 0.31 (range 0.17–0.45) in successful colonoscopies.

**Conclusion:**

This tool for evaluating the quality of colonoscopy procedures in healthcare units is based on standard endoscopy and patient criteria. It is an easy and feasible procedure giving the ability to detect suboptimal practice and differences between endoscopy-units. It will enable individual units to assess the quality of their colonoscopy techniques.

## Introduction

Colorectal cancer is a major cause of cancer mortality worldwide, with more than one million new cases diagnosed annually [1]. While there is no consensus on the optimal modality for a screening programme for this disease, colonoscopy is currently the most sensitive and specific screening test. One of the key aims of healthcare provision is to optimize patients' clinical outcomes. With this in mind, procedures have been investigated across all medical specialties to assess and improve patterns of practice and clinical outcomes, and particularly for risk-associated procedures such as colonoscopy [2], [3].

The colonoscopy technique requires extensive training and regular practice, with its success dependent upon a number of factors including correct caecum intubation, cleaning of the colon, careful mucosal inspection, and operator experience [4]. With the rapidly rising costs of healthcare and the need to rationalize spending, it is important to avoid costly repeat procedures, as in the cases of incomplete colonoscopy.

While colonoscopy is regarded as a gold-standard exploratory technique, there is a persistent difference in risk reduction for right- and left-sided cancer that might reflect a remaining higher percentage of missed adenomas in the right colon [5], [6], [7]. Recently, it has been shown that the protective effect of colonoscopy was lower than expected, especially for cancer located in the right colon [8]. In the right colon, 5% of the cancer and almost 10% of the polyps over 10 mm are missed [9]. Kaminski et al confirmed the importance of adenoma detection rate as an independent predictor of the risk of interval colorectal cancer after colonoscopy screening and considered it as major quality criteria [8].

Both the technique and the skill of each individual endoscopist should be evaluated regularly to minimize the risks of perforation and haemorrhage associated with the procedure. In view of the large number of operators, increasing demand for procedures, different quality criteria, and time needed to evaluate each endoscopist, this is unlikely to happen in practice. A global evaluation of multiple quality criteria, including the adenoma rate, offers a good method of assessing the quality of an endoscopy unit. We have developed, following the French National health care program, a straightforward and user-friendly procedure, which covers all phases of the endoscopy procedure [10]. The topic of the present study was to validate the applicability of the procedure in daily practice, giving physicians' the ability to detect areas for continuous quality improvement.

## Methods

In developing this procedure, we decided to follow some important guidelines. First the technique should be simple with a restricted number of criteria arbitrarily limited to ten. Second, all of the different stages of colonoscopy should be evaluated including informed consent from the patient, quality preparation, quality of the act, and risk factors for complications. Third, the technique should be reproducible, and evaluated in all endoscopy centers.

### Quality-control criteria

All patients give written informed consent for the study and the study was in accordance with Declaration of Helsinki and was approved by the Comité de protection des personnes d'île de France. Global evaluation is comprised of a checklist of 10 quality-control indicators covering the entire procedure of colonoscopy including pre-colonoscopic visits, co-morbidities and medications, the procedure itself, colonoscopy reports and anatomopathological reports. The observational studies were prospectively conducted and reported following the STROBE statement [11]. The list was designed to enable systematic data collection during the pre-colonoscopy visit or immediately before the procedure, and was complementary to items found in standard colonoscopy reports. The criteria and the procedure have been validated in a prior single-centre study [10]. Quality control indicators were validated by a panel of endoscopists (n = 9) to fulfill quality criteria and were compatible with the published literature and health authority guidelines [4], [12], [13]. They are thereby applicable in endoscopy units (Table 1, Table S1).

**Table 1:** Quality criteria for colonoscopy (N = 10).

Items independent of the colonoscopy procedure *(noted prospectively on colonoscopy checklists)*
1) Patient characteristics (specific information about colonoscopy risk determined by the gastroenterologist)
2) Informed consent about Creutzfeldt–Jakob disease
3) Comorbid conditions (valvulopathy)
4) Treatment with drugs which might increase the bleeding risk:
(Antiplatelets, heparin and Vitamin K antagonists)
5) Appropriateness of the colonoscopy indications (6 indications)
• Digestive haemorrhage
• Functional bowel disorder
• Screening colonoscopy
• Digestive symptoms refractory to symptomatic treatment
• Personal history of colon cancer or adenoma or inflammatory bowel disease
• Familial history of adenoma or colon cancer
Items dependent on the colonoscopy procedure *(included systematically in colonoscopy or pathology reports)*
6) Quality of the colonic preparation
7) Completeness of the procedure
8) Number of adenomas or adenocarcinomas found per procedure
9) Colonoscopy difficulty
10) Sedation

### Data collection

Gastroenterologists completed the colonoscopy checklist during the pre-colonoscopy visit or immediately before the procedure. After the procedure, the nurse placed the colonoscopy chart in a standard folder and added the pathology report later, thus minimizing the amount of work required to complete the quality assessment.

### Timetable

The multicentre feasibility study was done over a 12-month period, from 1 January 2008 to 1 January 2009. Data on 2000 procedures were collected at 10 centres in France, including two university hospitals, two general hospitals, five private practice centres, and one military hospital. The military hospital was considered as a general hospital. Two hundred consecutive colonoscopies were documented at each centre. Centres voluntarily participated in the study and were included if the endoscopy centre fulfilled more than 1000 colonoscopies per year. Endoscopists were included in the present study if they performed an endoscopy during the study. Three to 10 endoscopists per centre were evaluated.

### Quality indicators

We determined ten quality indicators for colonoscopy (table 1). Five of those items were independent of the colonoscopy procedure (Patients characteristics, informed consent about Creutzfeldt-Jakob disease, co-morbid conditions, treatment with drugs which might increase the bleeding risk, appropriateness of colonoscopy indications). Patients' characteristics consisted of the information given to the patients about the colonoscopy and about the risks. Informed consent about Creutzfeldt-Jakob disease was considered given if the patient received specific information from physicians. Co-morbid conditions included valvulopathy or other risk factors for endocarditis. The following drugs with a bleeding risk were reported: antiplatelets, aspirin, heparin and vitamin K antagonists. The appropriateness of the colonoscopy indications were considered in accordance with French guidelines [14]. Six validated indications were considered including digestive haemorrhage, functional bowel disorder, colonoscopy screening (following faecal blood tests), digestive symptoms refractory to symptomatic treatment, personal history of colon cancer or adenoma or inflammatory bowel disease and familial history of adenoma or colon cancer [12]. All others indications were considered as invalid indications.

Five of the chosen criteria were dependent on the colonoscopy procedure (quality of the colonic preparation, completeness of the procedure, adenoma or adenocarcinoma detection rate, colonoscopy difficulty, and sedation). The evaluation of effectiveness of various laxative regimens for bowel preparation was divided into 3 categories [15], [16]: “Good” is typically no or minimal solid stool with large amounts of clear fluid requiring suctioning. “Fair” refers to collections of semisolid debris that are cleared with difficulty. “Insufficient” refers to solid or semisolid debris that cannot be effectively cleared. Completeness of the procedure was identified by caecal intubation. Caecal intubation is achieved when the tip of the colonoscope has passed beyond the lip of the ileo-caecal valve into the caput coli, allowing the visualization of the medial walls of the caecum lining proximal to the ileo-caecal valve [17]. Colonoscopy difficulty was defined by the ease of caecal reaching. Colonoscopy was considered not easy if a technical difficulty was observed. The existence of a recent episode of diverticulitis, marked angulations, pelvic adhesions or stenosis was considered as risk factors for difficult colonoscopies. The adenoma detection rate was defined as the proportion of screened subjects in whom at least one adenomatous lesion was identified. An adenoma was considered advanced if it had a diameter >10 mm and/or villous and/or displayed the presence of severe dysplasia through histology. Sedation was defined as the use of general anaesthesia. General anesthesia for colonoscopy was done with total intravenous anaesthesia using Propofol. Photographic documentation was systematically performed.

### Statistical analysis

Statistical analysis and univariate statistical analysis were performed with Statview 5.0. To compare categorical and continuous variables between the groups, chi^2^ tests and analysis of variance (ANOVA), respectively, were used. A p value<0.05 was considered to be significant.

## Results

### Colonoscopy procedure

Of the 2000 procedures performed, 600 (30%) were done at general hospitals, 400 (20%) at university hospitals, and 1000 (50%) in private practices. The patients' characteristics are given in tables 2 and 3. All quality criteria have a less than 5% missing data rate in all centres except for Creutzfeldt-Jakob disease information (Table S1). The overall median age was 57.8 years (range: 43.7–71.9), and patients undergoing colonoscopies were women in 50.6% of the procedures. The colonoscopy was carried out for valid indications in accordance with French guidelines for 95.9% of the cases (range: 92.6–100). The colon preparation was reported to be insufficient in 3.7% of the procedures (range: 1–10.5). Caecal intubation rates were 95.3% (range: 81–99).

**Table 2:** Patient characteristics and procedural data (n = 2000).

	General hospital		University hospital		Private office	
		% MD**		% MD**		% MD**
N	600		400		1000	
Median age (years)	57.4	1	58.7	2.5	56.5	0.1
Sex Male/Female (%)	53/47	0	51/49	0.3	46/54	0.1
Prior colonoscopy (%)*	39.2	3.5	50.5	0	45.2	0.3
Prior colonoscopy results* normal (%)	48.0	-	50.5	-	40.9	-
Recognized indication for colonoscopy* (%)	94	2.5	93.1	2.5	98.2	0.7
Use of concomitant medications (%)	15.8	3.8	17.5	0.5	6.6	0.2
Personal history (%)		1.2		0.8		1.1
Colorectal cancer*	2.2		2.3		1.3	
Advanced adenoma*	3.2		6.3		4.0	
Adenoma* (advanced adenoma excluded)	11.5		14.3		10.6	
Polyps*	4.5		8.5		6.7	
Patient queried about Creutzfeldt–JaKob disease (%)	95.2	3,5	66.8	0,3	77.9	0.2
Oral lavage solution* (%)		0.2		1		0
Polyethylene glycol	65.2		50.3		71.5	
KLEAN PREP®	33.2		-		-	
FLEET®	0.6		-		24.1	
Unknown	1.0		49.7		4.4	
Preparation quality* (%)		0		1		0
Good/Fair	93.5		96		98.2	
Insufficient	6.5		5		1.8	
Sedation* (%)		0.2		1		0
General sedation	91.2		91.4		99.9	
Nitrous oxide	-		1.5		-	
None	8.8		7.1		0.1	
Colonoscopy difficulty* (%)		0		1		0
Difficult	10.2		11.0		2.2	
Easy	89.8		89.0		97.8	
Colonoscopy progression* (%)		0		1		0
Ileal intubation or reach caecum	91.2		93.8		98.1	
Incomplete	8.8		5.2		1.9	

* Expressed per 100 colonoscopies;

** % MD: percent of missing data.

**Table 3:** Patient characteristics and procedural data per centre (n = 2000).

	General Hospital			University Hospital		Private Office				
Centre	1	2	3	4	5	6	7	8	9	10
N	200	200	200	200	200	200	200	200	200	200
Median age (years)	56,5	61,3	55	59,9	57,5	56,2	57	58,7	56,4	-
Sex Male/Female (%)	56/44	57/43	46/54	50/50	53/47	46/54	48/52	44/56	44/56	-
Prior colonoscopy (%)*	49	68,2	36	57,8	43,5	33,7	50,5	52	48,7	42
Prior colonoscopy results* normal (%)	54,7	30,4	52,8	46	56,3	47,8	45,5	58,8	53,6	91,6
Recognized indication for colonoscopy* (%)	93,5	92,6	95,9	93,7	92,5	100	98	98,5	99,5	95
Use of concomitant medications (%)	9	15,2	23	17,2	18	5	7,5	7,5	5	8
Personal history (%)										
Colorectal*	1,5	2	3	2	2,5	3,5	1	1	0,5	0,5
Advanced adenoma*	2	4	4	10	3,5	7,5	6	4	4	0,5
Adenoma* (advanced adenoma excluded)	10,5	13,5	10,5	21,5	7	9,5	16	14	13	0,5
Polyps*	14	17	11,5	25,5	16	16	23	19	19,5	1
Patient queried about Creutzfeldt–JaKob disease (%)	95,3	92	98,3	75,9	57,5	92	68	72	92	62,4
Oral lavage solution* (%)										
Polyethylene glycol	95,5	0,5	100	99,5	0	100	7,5	89	91	70
KLEAN PREP®	0	99,5	0	0	0	0	0	0	0	0
FLEET®	1,5	0	0	0	0	0	76,5	8,5	8	27,5
Unknown	3	0	0	0,5	100	0	16	2,5	1	2,5
Preparation quality* (%)										
Good/Fair	97,5	89,5	93,5	93,5	94,9	96,5	99	98,5	98,5	98,5
Insufficient	2,5	10,5	6,5	6,5	5,1	3,5	1	1,5	1,5	1,5
Sedation* (%)										
General sedation	74,5	100	99	83,5	99,5	100	100	99,5	100	100
Nitrous oxide	0	0	0	3	0	0	0	0	0	0
None	25,5	0	1	13,5	0,5	0	0	0,5	0	0
Colonoscopy difficulty* (%)										
Difficult	3,5	21	6	17	5,1	4	0	1,5	0	5,5
Easy	96,5	79	94	83	94,9	96	100	98,5	100	94,5
Colonoscopy progression* (%)										
Ileal intubation or reach caecum	99	81	93,5	92	97,5	96,5	99	99	98	98
Incomplete	1	19	6,5	8	2,5	3,5	1	1	2	2

* Expressed per 100 colonoscopies.

### Colonoscopy success

The rate of successful colonoscopies was above the 90% recommended caecal intubation rate in all centres evaluated except centre 3. In the university hospitals (centre 4 and 5), 8.6% of patients undergo colonoscopy without sedation and there is a trend for a lower rate of successful colonoscopy in patients under sedation (96.4%) versus unsedated patients (91.2%; p = 0.14). Colonoscopies were considered difficult by physicians in 6.4% (range: 0–21) of the cases. The rate of successful colonoscopy was more than 95% in all centres except centre 3.

### Adenoma detection rate

**Figure 1:**
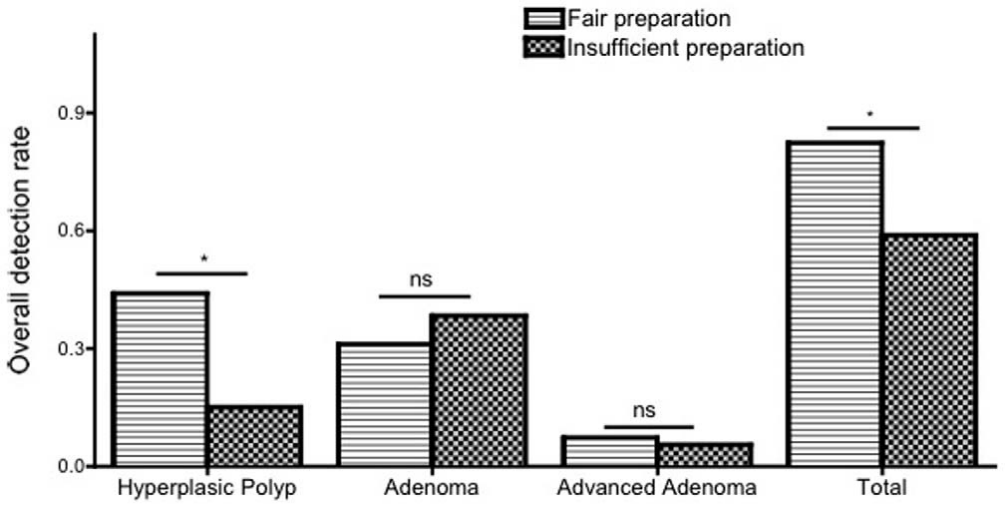
Overall detection rate in fair and insufficient preparation.

**Figure 2:**
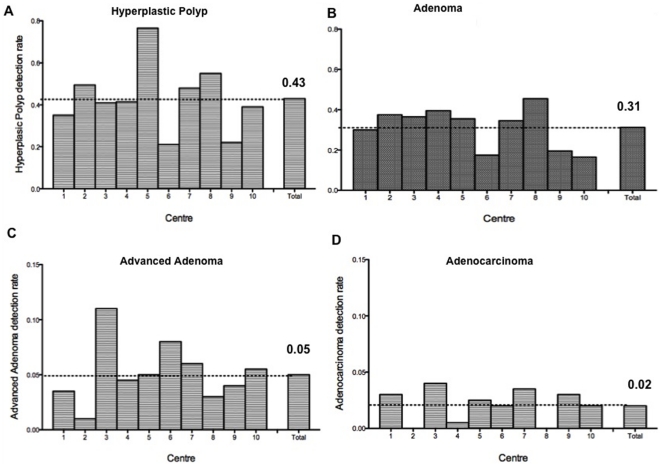
Endoscopic lesions detection rate: Hyperplastic polyps (a), Adenomas (b), Advanced adenomas (c), and Neoplastic lesions (d).

In patients with fair rated bowel preparations, overall lesion detection rate was higher than in poor bowel preparations (0.82 vs 0.59; p = 0.01) (Fig. 1). Neither the quality of the bowel preparation nor the difficulty of the colonoscopy was found to have a significant impact on adenoma detection rate and advanced adenoma detection rate. Adenoma detection rate was not significantly lower regarding the ability to reach the caecum (0.18 vs 0.14; p = 0.65) (Table 4). Adenoma or hyperplasic polyp detection rates were above 15% and 20% respectively in all centres (Fig. 2a,b). Carcinomas or advanced adenomas were diagnosed in respectively 2.0% and 4.8% of the colonoscopies (Fig. 2c, d). Adenocarcinoma detection rates were above 2% in all centres except centre 4

**Table 4:** Detection rates for adenoma and advanced adenoma and colonoscopy success.

	General hospitals	University hospitals	Private offices	Total
Adenomas				
- n	83	57	243	383
- %	13.8	14.3	24.3	19.2
Diagnosed if:				
- Successful colonoscopy (%)	14.5	14.4	29.5	17.4
- Unsuccessful colonoscopy (%)	7.5	14.2	22.3	14.4
Advanced adenomas or Adenocarcinomas, n (%)	29 (4.8)	25 (6.3)	36 (3.6)	90 (4.5)
Diagnosed if:				
- Colonoscopy successful (%)	4.4	6.1	3.6	4.5
- Colonoscopy unsuccessful (%)	21.4	7.0	21.1	18.4

Expressed per 100 colonoscopies.

## Discussion

Colonoscopy is widely used for colorectal cancer screening [18], [19], [20] Its miss rate for advanced adenomas, neoplastic lesions or adenomas remains a concern [21], [22], [23]. We report a multicentre, prospective, and time friendly method for evaluating the quality of professional practices in endoscopy units. In our study, widely recommended criteria for colonoscopy procedures were analysed, including adenoma detection rate, successful colonoscopy rate, and validated indications rate and bowel preparation quality. The European Panel on the Appropriateness of Gastrointestinal Endoscopy (EPAGE) multicentre study examined colonoscopy practices in endoscopy centres and pointed out wide variations in colonoscopy practices between centres justifying quality evaluation [24]. The feasibility of implementing the evaluation practices in all endoscopy units allows annual reviews. Therefore, it can be used to evaluate quality and improvements in performance at an individual unit level. This approach allows the evaluation of the whole endoscopy unit, thus enabling the quality of an individual unit to be assessed easily throughout the year. The next step might be a computer-based and integrated system with the colonoscopy report and the pathology database. In our study, we performed an overall evaluation procedure but our procedure required four different paper documents for each patient that need to be prospectively collected and then reviewed. Computer software will allow continually performing evaluations and also opening the doors for individual evaluations.

For some years, a trend of quality control appeared to improve colonoscopy procedures. In the past ten years, the rate of incomplete colonoscopy has declined from 19% to 10% [25] reflecting the publication of quality guidelines. Therefore, public health authorities have asked for feasible, simple, cost effective quality safety guidelines, driven towards effectiveness and improved public health. Following that, quality procedures should not only include the quality of the act, but also the performance, underlying diseases, and risk of certain medications to patients. The definition of high-quality colonoscopy and the factors with which to measure quality have been discussed [26], [27]. The recommended criteria include the use of appropriate screening and surveillance intervals, acceptable complication rates, appropriate caecal intubation rates, documentation of the quality of bowel preparation, and adenoma detection rates [12]. Predictive factors of incomplete colonoscopy include prior abdominal or pelvic surgery, practice variables, and physician variables, with notable differences reported between practice types (i.e., private office, academic, community hospitals), physician specialties, and colonoscopy volumes [25]. Evaluation of defined quality indicators will raise the quality of healthcare provisions in terms of safety, quality, and adequacy of bowel preparation, leading to better patient outcomes in direct response to the increasingly stringent demands from healthcare recipients and professional societies. Quality indicators for colonoscopy have been selected to establish competence in performing colonoscopy procedures and to help define areas for continuous quality improvement [12], [13], [24]. No procedure for colonoscopy evaluations is available yet at the European or American level. In the present study, chosen quality criteria are in accordance with validated quality indicators for colonoscopy and the procedure will help endoscopy units define areas for continuous quality improvement.

In the 2000-colonoscopy procedures reviewed here, the adenoma detection rate was 19%. These data are in accordance with published data [10], [28] and illustrate the need for good clinical practice to ensure accurate and early detection, and hence treatment, of adenomas. The rate of adenoma detection is strongly associated with the quality of the colonoscopy. Risk of intervalic cancer was significantly higher among subjects who underwent colonoscopies that were performed by endoscopists with an adenoma detection rate of less than 20% than among subjects examined by endoscopists with a detection rate of 20% or more [8]. Under highly standardized conditions, colonoscopy has been associated with a significant reduction in the incidence of both left- and right-sided colorectal cancer [29]. Interestingly, in our study, the adenoma detection rate was under 20% in half of the units. Our study illustrates the usefulness of a validated assessment protocol to evaluate the quality of the overall procedure and highlights some procedural deficiencies. Taking this into account, physicians should repeat the evaluation procedure every year and hopefully confirm the improvement of the procedure. A 20% adenoma detection rate should be a baseline criterion to consider a centre as an expert centre.

The annual risk of cancerous transformation for adenoma, macro adenoma (above 10 mm), and high-grade dysplasia adenoma are 0.25%, 3% and 37% respectively [30]. The advanced adenoma detection rate might be a useful quality indicator in colonoscopy. Adenoma detection rates among experienced colorectal physicians vary widely [26], [31]. The advanced adenoma detection rate in our study was between 3.6% in private practices and 6.3% in university hospitals. On the basis of the prevalence of advanced adenomas (4.8% to 9.7%) [19], [20], The threshold values for rates of advanced adenoma detection should be between 5 to 10%. There is no proof that these values apply to large-scale screening programs involving centers with lower adenoma detection rates. Nevertheless, the risk of colorectal cancer after a previous negative colonoscopy is very low. Brenner et al recently highlight that a substantial proportion of interval cancers are due to neoplasms missed at colonoscopy and are potentially preventable by enhanced performance of colonoscopy [32]. Those conclusions play for quality indicators in colonoscopy to reduce colorectal cancer risk.

Adenoma detection rate is a validated quality criterion and is hardly evaluable in a centre. Recently published data estimated a range of adenoma detection rates from 11%–28% [8], [33], [34].Taking this into consideration, the desired adenoma detection rate in each centre must be above 15%. This rate should be adjusted in each centre according to the population and in accordance with the physicians. Therefore, a validated evaluation procedure, undertaken each year is necessary to improve both quality criteria and self-assessment.

The quality of the bowel preparation can impact adenoma detection [35]. In our study, the number of insufficient colonic preparations was ranged from 1.8% in private practice to 6.5% in general hospitals. Endoscopists in private practice appear to invest more time in ensuring the quality of the bowel preparation, or patients who undergo colonoscopy in private centres are more concerned about its importance. This might explain their high rates of good quality colonic preparation and successful colonoscopy. On the other hand, in our study notable differences exist between patients in private practices and other centres. Patients attending private offices were mainly female (74%) and the rates of complete colonoscopy were higher, 98.1%, versus 93.8% in university hospitals and 91.2% in general hospitals. Educating the patient and providing them with clearly written information about the importance of optimal bowel preparation will improve the likelihood of a successful procedure.

Dirty bowel preparation increases the number of false-negative results and is a predictive factor of incomplete colonoscopies (OR: 11,957; 9,085-15,740) [36]. The number of incomplete colonoscopies did appear to correlate directly with the number of insufficient colonic preparations [36]. In a European multicentre study, Harris et al. pointed out that there are wide variations in high-quality cleansing of patients (range 51–94%) between centres [24]. Our study showed that a relatively high proportion of colonoscopies are completed with insufficient or fair bowel preparation (20.4%) compromising the operator's ability to detect adenomas. Among the 2000 colonoscopies, polyp detection was significantly higher with fair preparation than with less than fair preparation (36.8% versus 20.5%; p = 0.012; RR = 0.56. IC95% = 0.35–0.88). Also, in our study, we showed an apparent, yet insignificant tendency for lower adenoma detection rates with less than fair preparations. (19.3% versus 15.1%; p = 0.375).

Public health authorities recommended registering comorbid conditions and risk factors such as Creutzfeldt-Jakob disease [37]. In our study, this criterion was considered as a quality criterion. Objective evidence of surgical transmission of sporadic Creutzfeldt-Jakob disease remains debatable in part due to the misclassification of exposure levels [38]. Prion neuro-invasion is likely to represent a causal relationship between surgery and a non-negligible proportion of sporadic Creutzfeldt-Jakob disease cases. Endoscopic transmission of prion-mediated infectious diseases has never been described. Nevertheless, in affected patients, the Creutzfeldt-Jakob disease prion is present in the tonsils and the digestive tract. The Creutzfeldt-Jakob disease information criterion has been chosen to reinforce the importance of informing and educating physicians to systematically give information to patients about Creutzfeldt-Jakob disease transmission risks.

Non-sedated colonoscopy is not a pleasant experience for patients. Non-sedated patients would certainly be less likely to tolerate a complete examination. In usual practice, complete colonoscopy are strongly associated with fewer deaths from colorectal carcinoma even is the association is limited to deaths from colorectal carcinoma in the left side of the colon [39]. Patient discomfort was identified as a reason for incomplete colonoscopy in 15.3% of the cases [40]. Sedation offers the patient a greater degree of comfort and facilitates the procedure for the practitioner. In our study, the rate of sedation varied from 91.2% in general hospitals to 99.9% in private practices. Analgesia during colonoscopies has been validated for both completeness and lowering of the risk of acute complications [36]. Our study did not confirm these data, as there was no increase in the rate of incomplete colonoscopies without analgesia. The majority of patients undergoing colonoscopy under sedation causes a lack of power and limits the interpretation of this quality criterion.

The evaluation forms as reported in the development of the procedure included fifteen items and 4 intra or post procedural items [10]. In our study, we arbitrarily limited the number of items to ten in order to avoid high percentages of missing data. Therefore, 5 items focusing on endoscope washing, procedures with tissue samples, and tracking sheets were not considered in the present study. Missing data dropped from the pilot study to the present study from 56% to less than 5%.

We report the first multicentre prospective evaluation of the quality of colonoscopy procedures in healthcare units. This technique incorporates a checklist of 10 quality criteria and requires no major changes in practice. This procedural evaluation helps centres to identify suboptimal practices and differences between endoscopy-units in sufficient bowel preparation rate, successful colonoscopy rate and adenoma detection rate. It is an easy and feasible procedure across all endoscopy centres. The evaluation can be done at any point in time, and takes a minimal amount of time to complete. By using this technique, individual centres will be able to assess and improve their performances, so that colonoscopy remains an uncontested gold standard in screening for colorectal cancer.

## Supporting Information

Table S1 Missing data rate per criterion (n = 2000).
(DOCX)
