# Supplementary material for: Quality Indicators for Colonoscopy Procedures: A Prospective Multicentre Method for Endoscopy Units
Source: PLoS One. 2012 Apr 11;7(4):e33957. doi: 10.1371/journal.pone.0033957 (PMC3324486; doi:10.1371/journal.pone.0033957)
Supplement: Table S1 — Missing data rate per criterion (n = 2000). (DOCX) [file pone.0033957.s001.docx]

Table S1: **Missing data rate per criterion (n=2000)**

|  | **General hospital** | | | **University hospital** | | **Private office** | | | | | **Total** |
| --- | --- | --- | --- | --- | --- | --- | --- | --- | --- | --- | --- |
| **Centre** | **1** | **2** | **3** | **4** | **5** | **6** | **7** | **8** | **9** | **10** |  |
| N | 200 | 200 | 200 | 200 | 200 | 200 | 200 | 200 | 200 | 200 | 2000 |
| Median age (%) | 1 | 2 | 1 | 3 | 3 | 1 | 1 | 1 | 100* | 2 | 11.5 |
| Sex Male (%) | 1 | 2 | 1 | 3 | 3 | 1 | 1 | 1 | 100* | 2 | 11.5 |
| Patient queried about Creutzfeldt–Jakob disease (%) | 5 | 10 | 11 | 1 | 40 | 3 | 3 | 2 | 5 | 5 | 8.5 |
| Comorbid condition (valvulopathy) (%) | 3 | 2 | 1 | 1 | 2 | 1 | 2 | 1 | 1 | 1 | 1.45 |
| Use of concomitant medications (%) | 1 | 3 | 1 | 1 | 3 | 1 | 1 | 1 | 1 | 1 | 1.4 |
| Appropriateness of the colonoscopy indication (%) | 3 | 3 | 5 | 5 | 4 | 5 | 1 | 3 | 4 | 3 | 3.6 |
| Preparation quality (%) | 0 | 1 | 1 | 0 | 2 | 1 | 0 | 0 | 1 | 0 | 0.6 |
| Colonoscopy progression (%) | 2 | 1 | 2 | 2 | 2 | 1 | 1 | 1 | 1 | 0 | 1.3 |
| Histopathological results (%) | 0 | 1 | 0 | 0 | 1 | 1 | 2 | 2 | 1 | 0 | 0.8 |
| Colonoscopy difficulty (%) | 2 | 1 | 1 | 0 | 2 | 1 | 0 | 1 | 2 | 2 | 1.2 |
| Sedation (%) | 0 | 1 | 0 | 1 | 2 | 1 | 0 | 0 | 1 | 0 | 0.6 |

* Patients’ personal data (sex and age) were voluntarily censored by centre.
